# A Promising Thumb Basal Joint Hemiarthroplasty for Treatment of Trapeziometacarpal Osteoarthritis

**DOI:** 10.1007/s11999-012-2367-7

**Published:** 2012-05-15

**Authors:** James W. Pritchett, Louis S. Habryl

**Affiliations:** 1 Orthopedics International, Seattle, WA USA; 2 Department of Orthopedic Surgery, University of Washington, 901 Boren Avenue, #800, Seattle, WA 98104 USA; 3 N’Orthopedics, PC, Gaylord, MI USA; 4 Department of Orthopedics, Midwestern University, Gaylord, MI USA

## Abstract

**Background:**

Trapeziometacarpal joint osteoarthritis is a painful, disabling condition that primarily affects women who are postmenopausal. Arthroplasty has been performed to treat this condition; however, subluxation has been a problem with all previous implants. We report the results of hemiarthroplasty using a prosthesis designed to address the problems associated with previous implants.

**Questions/purposes:**

We wished to (1) determine if this prosthesis results in pain relief and functional improvement and preserves the appearance of the thumb, (2) assess the prosthetic reconstruction during followup, (3) assess complications that occur with the use of this prosthesis, and (4) determine the survivorship of this prosthesis.

**Methods:**

We performed 159 basal joint hemiarthroplasties (138 patients) to treat osteoarthritis of the trapeziometacarpal joint. The mean age of the patients was 63 years, 78% were women, and all had Eaton-Littler Stage II or III changes. Only the damaged articular surfaces of the metacarpal and trapezium were excised; no tendon grafts or transfers were performed. Seven patients (seven thumbs) were lost to followup and seven (nine thumbs) died, leaving 124 patients (143 thumbs) for review. Clinical and radiographic assessments were made preoperatively, 12 weeks postoperatively, and annually thereafter. Minimum followup was 35 months (mean, 72.1 months; range, 35–120 months).

**Results:**

At latest followup, pain relief occurred in 135 thumbs, function improved in 138 thumbs, 139 thumbs were excellent or good in overall assessment, and 142 thumbs had good or excellent cosmetic appearance. The mean tip pinch improved from 4.9 kg preoperatively to 6.44 kg postoperatively. Mean postoperative Buck-Gramcko score was 49 (excellent); overall Kaplan-Meier analysis with revision as the end point showed 94% implant survivorship at a mean followup of 72.1 months.

**Conclusions:**

Our results are superior to those of other implants and support continued use of this implant. Studies with longer followup are required to confirm these results.

**Level of Evidence:**

Level IV, therapeutic study. See the Guidelines for Authors for a complete description of levels of evidence.

## Introduction

Osteoarthritis of the trapeziometacarpal joint predominantly afflicts women who are postmenopausal [2, 7, 21, 22]. The cartilage and fibrous tissues at the base of the metacarpal have been found to bind relaxin-related compounds [21]. Progressive deterioration of the beak ligament of the trapeziometacarpal joint leads to joint instability and cartilage deterioration [19, 21, 22]. The cause of this deterioration is not known, but the predominance of trapeziometacarpal arthritis in women suggests hormones play an important role.

Most patients with basal joint arthritis choose to live with the condition and avoid thumb abduction and strong key pinch movements. For many patients, however, this painful and disabling condition interferes with activities of daily living, job performance, and recreational pursuits. Deformity is variable and can include adduction of the metacarpal shaft and metacarpophalangeal joint hyperextension or subluxation [7, 21].

All cases, regardless of severity, are treated initially with nonoperative measures consisting of medication, splinting, physical therapy, and injections. When nonoperative treatment fails, several surgical options are available. Several treatments have been offered, but none is uniformly successful [1, 3, 13, 20]. Trapeziometacarpal arthrodesis has been used occasionally, yet a solid radiographic union does not always occur and reported nonunion rates range from 5% to 50% [6, 11]. In addition, arthrodesis transfers stress to adjacent and possibly diseased joints and the loss of motion can make it difficult to place the hand into restricted areas and on flat surfaces.

For moderately severe disease, ligament reconstruction or osteotomy can be effective. For severe disease, treatment options include arthrodesis, excision, or tendon interpositional arthroplasty [6, 11, 14–16]. Excisional arthroplasty was introduced in 1949 according to Ferrari and Steffee [13]. Froimson [14] reported the use of the rolled flexor carpiradialis “anchovy” spacer after total trapeziectomy. Although pain relief was universal, he described a 30% reduction in pinch strength and 50% loss of arthroplasty “space” secondary to metacarpal settling after 6 years of followup. Hemitrapeziectomy was later suggested [15] with the hope of minimizing thumb shortening and improving pinch strength. Various ligament reconstructions can be added to support the interpositional procedure. Other authors have suggested the use of other tendons, fascia lata, banked cartilage, and Gelfoam^®^ as interpositional materials [10, 22]. Postoperative weakness and instability are the drawback of these operations.

Implant arthroplasty has been performed for 45 years using various types of implant designs and materials, such as silicone, ceramic, polyethylene, and metal [1, 3, 4, 13, 18, 20, 24–26]. Swanson [24, 25] primarily was responsible for popularizing silicone implant arthroplasty. Swanson reported excellent pain relief and functional improvement; however, 20% of patients experienced subluxation of the prosthesis. A high incidence of silicone synovitis led to the abandonment of this prosthesis [8]. In 1981, Swanson et al. [26] began using a titanium implant, but subluxation still occurred in several patients. Implant subluxation and high failure rates have been a problem with all previous prosthetic designs and implant materials [3, 18, 20, 25].

**Fig. 1:**
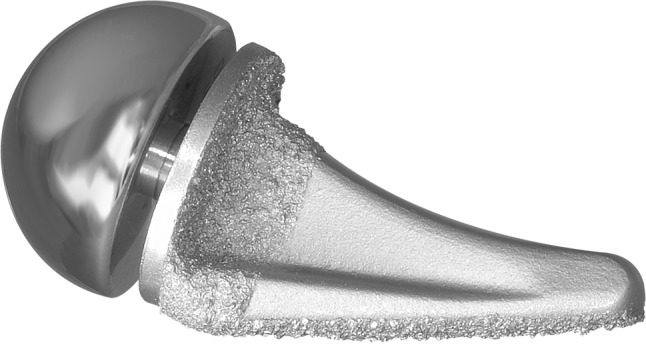
A photograph shows the BioPro^®^ Modular Thumb. (Photograph supplied by and published with permission from BioPro, Port Huron, MI, USA.)

A metal, stemmed, basal joint prosthesis for hemiarthroplasty has been introduced to treat patients with Eaton-Littler Stages II and III trapeziometacarpal arthritis (BioPro^®^ Modular Thumb; BioPro, Port Huron, MI, USA) (Fig. 1). The stem is porous coated and has a titanium plasma spray for cementless fixation. The design features of this implant address the aforementioned problems associated with previous prosthetic designs. First, this implant features a varus (adduction) angle to replicate the normal orientation of the trapeziometacarpal joint by placing the thumb metacarpal in the desired relationship to the hand; most previous implant designs placed the articular surface perpendicular to the stem. The second feature is modularity, which allows the size of the convex head of the implant to be adjusted independently of the size of the metacarpal stem. This is particularly beneficial for older women who commonly have a large metacarpal medullary space yet a small trapezium. The modular head also allows additional adjustment of ligament tension by adding length choices at the trunion.

This report describes our results using this implant design. Our aims were to (1) assess pain relief and functional improvement and preservation of the appearance of the thumb as reflected by improved Buck-Gramcko scores [5]. (2) We also wished to radiographically assess the prosthetic reconstruction during followup and (3) document what complications occur with the use of this prosthesis. (4) Finally we wished to calculate the Kaplan-Meyer survivorship of this prosthesis using need for revision as the end point.

## Patients and Methods

Institutional review board approval was obtained and all participants provided written informed consent. This is a prospective single-cohort study consisting of 124 selected patients (143 thumbs) with Eaton-Littler Stage II or III osteoarthritis of the trapeziometacarpal joint who underwent thumb basal joint hemiarthroplasty using the BioPro^®^ Modular Thumb prosthesis between 2001 and 2008. The indications for surgery were difficulties in performing work duties or activities of daily living because of basal joint thumb pain. Inclusion criteria were Eaton-Littler Stages II and III trapeziometacarpal involvement. Exclusion criteria were Eaton-Littler Stage IV (because treatment consists of excising the entire trapezium and a joint resurfacing prosthesis such as the one described in this report is not appropriate), pantrapezial arthritis, posttraumatic deformity, and inflammatory arthritis (one of us [JWP] would perform this implant arthroplasty for an old Bennett’s fracture, depending on the degree of deformity, but this was not part of the indications during this study). During the period of this study, 27 of the 165 eligible patients met the exclusion criteria (18 attributable to Eaton-Littler Stage IV disease, one attributable to prior infection, four attributable to inflammatory arthritis, and four attributable to posttraumatic deformity or prior fusion) and were offered other procedures and treatments, thus leaving 138 patients (159 thumbs) eligible to participate. There were 107 women (mean age, 62 years; range, 41–85 years; SD, 9 years) and 31 men (mean age, 65 years; range, 50–77 years; SD, 8 years). Seven patients (nine thumbs) died and seven patients (seven thumbs) were lost during the followup period. Therefore, data are reported for 124 patients (94 women, 30 men; 143 thumbs). The minimum followup was 35 months (mean, 72.1 months; range, 35–120 months).

Previously, all patients had undergone nonoperative treatment consisting of exercises, splints, and antiinflammatory medication. Patients had symptoms for a mean of 38 months before surgery (range, 18–60 months). The Eaton-Littler classification system [12] has been used to stage thumb carpometacarpal joint osteoarthritis radiographically (Table 1). Only more recently has the reliability of the system been evaluated. In 2002, Kubik and Lubahn [17] used posteroanterior and lateral radiographs of the thumb carpometacarpal joint of 40 patients for evaluation by three orthopaedic surgical residents and three experienced hand surgeons on two separate occasions. Using κ statistical analysis, they classified the results as poor (0–0.50), moderate (0.51–0.75), or excellent (> 0.75). Overall, the intrarater and interrater reliabilities were moderate (mean, 0.657 and 0.529, respectively). Another reliability study used 40 sets of a combination of three radiographic views (posteroanterior, lateral, and Bett’s [Gedda’s] views) [9]. The authors noted an improvement in reliability when all three views were used, leading to intrarater reliability rated as good and interrater reliability as moderate. The most recent study showed 40 radiographic cases of the first carpometacarpal joint independently to five experienced musculoskeletal radiologists and eight hand surgeons [23]. All were asked to assign the Eaton-Littler stage and the hand surgeons also were asked for their preferred treatment (ie, nonoperative, ligament reconstruction or extension osteotomy, hemitrapeziectomy with interposition, arthrodesis, trapeziectomy, hemiarthroplasty, or total arthroplasty). Overall, the radiographic classification was rated as moderate, whereas the hand surgeons’ treatment selections were rated as fair. Dela Rosa et al. concluded such variance in classification and treatment warrant evidence-based research to improve classification and treatment of first carpometacarpal arthritis [9].

**Table 1:** Classification system of Eaton and Littler [12]

Stage	Characteristic radiographic findings
I	Synovitis phase; no significant capsular laxity; slight widening of the joint space due to effusion, normal articular contours, and < 1/3 subluxation in any projection
II	Significant capsular laxity, possibly at least 1/3 subluxation of the joint; instability apparent on stress radiographs; small bone of calcific fragments < 2 mm, usually adjacent to the volar or dorsal facets of the trapezium
III	< 1/3 subluxation, fragments > 2 mm dorsally or volarly, usually in both locations; slight joint space narrowing
IV	Advanced degenerative changes; more joint collapse than sclerosis and osteophyte formation present; major subluxation and very narrow joint space, with cystic and sclerotic subchondral bone changes; trapezial margins showing lipping and osteophyte formation; significant erosion of the dorsoradial facet of the trapezium

Adapted from and published with permission from Eaton RG, Littler JW. Ligament reconstruction for the painful thumb carpometacarpal joint. *J Bone Joint Surg Am.* 1973;55:1655–1666.

The surgical procedure began by exposing the thumb trapeziometacarpal joint using a slightly curved, dorsolateral incision. The capsule of the trapeziometacarpal joint was incised longitudinally with sharp subperiosteal dissection, with care taken to retain the capsule’s integrity with the exposure. The capsular attachments to the trapezium and base of the metacarpal, including a limited portion of the insertion of the abductor pollicis longus tendon, were released circumferentially. The trapeziometacarpal joint was exposed at the base of the thumb metacarpal parallel to the articular surface. Only a minimal resection of the damaged articulation was required. The base of the metacarpal was resected parallel to the varus-positioned articular surface (12°–15° in the sagittal plane). Access to the longitudinal axis of the metacarpal was facilitated by adduction and flexion of the thumb. The canal was opened and the canal broaches were inserted. The medullary cancellous bone was compressed progressively until a tight medullary interference fit was achieved. Sizing templates were used to estimate the size of the prosthetic head. The trapezium was prepared with a medium-sized bone burr. Care was taken to medialize the prepared concentric cavity to the adjoining facet with the index metacarpal. Reamers of different sizes were used to prepare the socket. The depth of the socket had to be sufficient to provide stable articulation during a trial reduction with the thumb adducted. The implant is available in four stem and head sizes that are interchangeable. The trial head and stem were implanted and reduced and the prosthetic articulation was tested for joint stability and freedom of movement. If the thumb could not be brought to 60° abduction without undue force, the joint was overstuffed and had to be revised by removing additional bone from the metacarpal or deepening the socket; the modular head and stem were selected with this in mind. A careful capsular closure that sometimes included placing a suture for the beak ligament was performed. The wound was closed with nylon sutures and a splint was applied. Three weeks postoperatively, the sutures and splint were removed and the patients began ROM exercises, with an emphasis on abduction and opposition. Full activity was allowed 6 weeks postoperatively.

We collected outcome measure data prospectively. Radiographic measurements and function assessments were made preoperatively, 12 weeks postoperatively, and annually thereafter. We assessed overall long-term outcomes using the Buck-Gramcko score [5], a standardized outcome measure that provides objective (palmar abduction, radial abduction, tip pinch) and subjective (pain frequency, strength, daily function, dexterity, cosmetic appearance, willingness to undergo the surgery again, overall assessment) data. A total score of 49 to 56 points (the maximum score) was rated as excellent; 40 to 48 points good; 28 to 39 points fair; and less than 28 points poor. A physician’s assistant (SS) skilled in the evaluation of thumb and hand function measured the objective parameters. Patients rated the subjective outcomes of the score by completing a self-administered questionnaire. Patients’ also subjectively assessed their abilities to perform specific functional tasks (write, brush teeth, turn keys, open tight jars, use scissors, buttons, zippers, pick up small objects, play/deal cards) using the surgically treated hand. Result data reported are based on the most recent postoperative followup assessments and radiographs.

We analyzed the data using the Wilcoxon signed-rank test or Student’s paired t-tests for normally distributed data. The Kaplan-Meier method was used for survivorship analysis with revision as the end point. All analyses were performed using statistical software (JMP^®^ Pro 9.0; SAS Institute Inc, Cary, NC, USA).

## Results

Basal joint arthroplasty using the BioPro^®^ Modular Thumb prosthesis resulted in decreased pain, improved function, and good appearance of the thumb as measured by the Buck-Gramcko score in this series of patients (p < 0.0001). The tip pinch strength was, on average, 1.5 kg greater (p < 0.0001) in patients after surgery as compared with preoperative values. Patients experienced an average increase of 3.6 points in radial abduction postoperatively (p < 0.0001). Eighty-nine percent of the patients could oppose the tip of the surgically treated thumb to the base of the small finger and all patients could oppose the thumb to all four fingers. All patients experienced an increase in the Buck-Gramcko score postoperatively, with an average change greater than 25 points (p < 0.0001) (Table 2). The mean preoperative Buck-Gramcko score increased 53% from 23 to 49 (excellent) postoperatively. In assessing overall outcomes, patients rated 139 thumbs as excellent or good, 135 thumbs had no or only occasional pain, and 138 thumbs had good or excellent functional improvement (Table 3). The results of patients’ subjective assessment of their abilities to perform specific functional tasks are presented (Table 4). Eleven patients had been treated previously with a tendon interposition on the contralateral thumb (nine of the 11 patients had Eaton-Littler Stage II and two had Eaton-Littler Stage IV disease). All of these patients preferred the implant thumb to the tendon interposition thumb.

**Table 2:** Objective outcomes for 143 thumbs as assessed according to the Buck-Gramcko score at most recent followup

Movement	Number of points	Number of thumbs
Palmar abduction		
≥ 40°	6	57
30°–39°	4	75
20°–29°	2	11
< 20°	0	0
Radial abduction		
≥ 40°	6	54
30°–39°	4	80
20°–29°	2	9
< 20°	0	0
Tip pinch compared with normal contralateral side		
> 100%	6	1
80%–99%	4	35
60%–79%	2	106
< 60%	0	1

**Table 3:** Subjective outcomes for 143 thumbs according to the Buck-Gramcko score at most recent followup

Characteristic	Number of points	Number of thumbs
Pain frequency		
Never	6	66
Occasional	4	69
Frequent	2	6
Constant	0	2
Strength		
Improved	6	89
Same	3	51
Worse	0	3
Daily function		
No difficulty	6	107
Mild difficulty	4	31
Moderate difficulty	2	4
Severe difficulty	0	1
Dexterity		
Improved	6	109
Same	3	31
Worse	0	3
Appearance		
Excellent	4	109
Good	3	33
Acceptable	2	1
Poor	0	0
Would you have surgery again?		
Yes	4	139
No	0	4
Overall assessment		
Excellent	6	85
Good	4	54
Fair	2	1
Poor	0	3
Grade of total score		
Excellent	49–56	68
Good	40–48	66
Fair	28–39	8
Poor	< 28	1
Mean total score	49

**Table 4:** Patients’ subjective assessment of their abilities to perform functional tasks using the surgically treated hand

Task	Number of thumbs					
	Total	No difficulty	Mild difficulty	Moderate difficulty	Severe difficulty	Unable
Write	143	127	10	6	0	NA
Brush teeth	143	135	4	4	0	0
Turn keys	143	92	45	6	0	0
Open tight jars	143	65	47	22	3	6
Use scissors	143	90	36	15	2	0
Buttons	143	99	37	5	1	1
Zippers	143	101	37	5	0	0
Pick up small objects	143	118	20	4	1	0
Play/deal cards	143	129	11	2	0	1

NA = not applicable.

**Fig. 2A–E:**
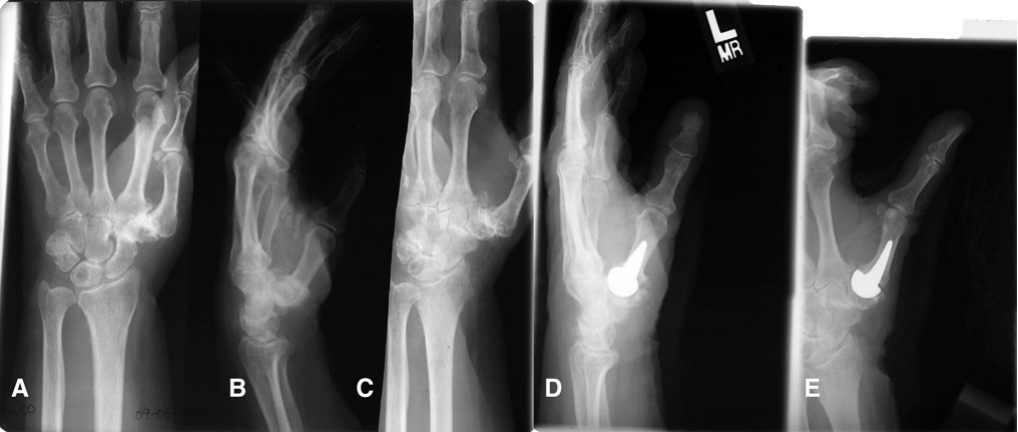
Preoperative (**A**) posteroanterior, (**B**) lateral, and (**C**) oblique radiographs show the left hand and wrist of a 63-year-old woman with Eaton-Littler Stage III arthritis of the basal joint of the thumb. (**D**) Lateral and (**E**) posteroanterior radiographs show the left hand and wrist 72 months after implant arthroplasty of the left thumb basal joint.

The radiographic assessment of the prosthetic reconstruction found all thumbs had remodeling at the base of the first metacarpal around the intramedullary stem and around the convexity of the implant head during the first 6 months after surgery and then remained stable (Fig. 2). The metacarpal length was preserved in all cases with the metacarpal shaft accommodating the shape of the implant. Bone deposition was observed around the porous-coated intramedullary stem indicating osseointegration. There was no evidence of stem migration, fracture, or heterotopic bone formation. There were 15 thumbs with peritrapezial arthritis.

There were few complications with this procedure. There was one intraoperative fracture that healed, one painful neuroma, and one infection. One patient had numbness over the radial nerve and arthritis elsewhere in the affected hand. One patient had 4 mm of erosion into the trapezium without symptoms 8 years after surgery. Deepening of the trapezial concavity (approximately 2 mm) occurred in seven thumbs during the first postoperative year and then remained stable.

**Fig. 3:**
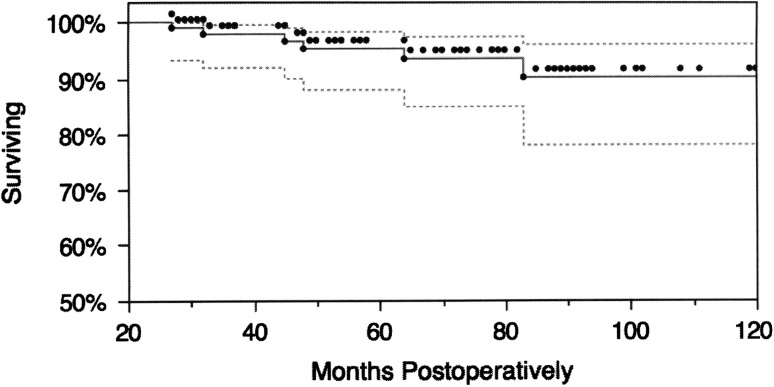
Overall Kaplan-Meier analysis with revision as the end point shows an implant survivorship of 94% at a mean followup of 72.1 months. Dotted lines = 95% CIs.

Overall Kaplan-Meier analysis with revision as the end point showed an implant survivorship of 94% at a mean followup of 72.1 months (Fig. 3). There were six revision procedures. One prosthesis was removed owing to pain 27 months postimplantation and the patient underwent tendon interposition with trapeziectomy yet continued to have pain. One prosthesis was revised successfully 29 months postimplantation for joint instability to a larger modular head; this patient reported instability starting at 6 months postoperatively but did not elect to undergo surgery until 29 months postoperatively. The other four successful revisions were for stem loosening (failure of ingrowth). No implant dislocated completely. The four patients with loosening had pain that seemed to be greater before and after applying pinch. The radiographs were fairly unremarkable and the symptoms were confirmed by surgical findings at revision.

The mean operating time was 44 minutes. The mean stem size was 9.21 mm (range, 7.5–12 mm) and the mean ball size was 13.08 mm (range, 12–15 mm). According to the patients’ subjective reports of their postoperative ability to perform functional tasks, the mean recovery time was 12 weeks.

## Discussion

Trapeziometacarpal joint osteoarthritis is a painful, disabling condition that primarily affects women who are postmenopausal. Arthroplasty has been performed to treat this condition; however, subluxation has been a problem with all previous implants. We report the results of hemiarthroplasty using a prosthesis designed to address the problems associated with previous implants. Our aims were to (1) assess whether this prosthesis results in pain relief and functional improvement and preserves the appearance of the thumb as reflected by improved Buck-Gramcko scores; (2) radiographically assess the prosthetic reconstruction in followup; (3) document what complications occur with the use of this prosthesis; and (4) calculate the Kaplan-Meyer survivorship of this prosthesis using need for revision as the end point.

Our study has some limitations. The implants were placed by experienced surgeons and the technique may not be mastered easily by others. Longer followup and a larger patient cohort would be desirable, although our followup greater than 6 years is adequate to detect any implant-related radiographic and functional problems and a 143-thumb cohort is much greater than that of other published studies. Another possible limitation is that only patients with osteoarthritis were included in the study. Thus, our results may not apply to the use of this implant and technique in treating posttraumatic arthritis, inflammatory arthritis, or other indications.

Postoperatively, our patients experienced significant pain relief and improved function as measured by the Buck-Gramcko score. Overall, patients subjectively rated 139 of the 143 thumbs as excellent or good, 136 thumbs had no or occasional pain, and 138 had good or excellent improvement in function. These results represent an improvement over results achieved with previous prosthetic designs. The experience of DeHeer et al. [8] and Swanson [24, 25] with a silicone rubber prosthesis resulted in pain relief and functional ROM but ultimately was unsuccessful owing to a 20% rate of subluxation and high incidence of silicone synovitis. Subluxation remained a problem with later use of a titanium implant [26]. Other investigators have found various other procedures that attempted to alleviate basal joint arthritis pain and improve function also ultimately were unsuccessful [1, 3, 13, 20, 24, 25].

We found our aims of preserving the cosmetic appearance and length of the thumb were achieved, as assessed by the Buck-Gramcko score and radiographic followup. Metacarpal length was preserved in all thumbs and the metacarpal shaft accommodated the shape of the implant well. Total trapeziectomy has been shown to provide pain relief; however, Froimson [14] reported a 50% loss of arthroplasty space attributable to metacarpal settling after 6 years of followup. In our study, all 143 thumbs retained their cosmetic appearance and length at a similar followup of 6 years.

We encountered few complications with this prosthesis (one intraoperative fracture, one neuroma, one infection, and six revisions) and there were no dislocations. As stated previously, subluxation has been the primary complication with earlier efforts and different prostheses; there were two subluxations in our series.

Finally, survivorship analysis found 94% of these prostheses were functional at a mean followup of 72.1 months (range, 35–120 months). This shows improvement over previous prosthetic designs that had high failure rates, such as silicone, ceramic, cemented (in younger, active patients), and carbon fiber [1, 3, 13, 18, 20, 24, 25].

We performed basal joint hemiarthroplasty to treat Eaton-Littler Stages II and III trapeziometacarpal osteoarthritis using an implant designed to address the problems associated with previous implant devices, namely, subluxation. Efforts to reduce the incidence of subluxation previously involved ligament augmentation, but this procedure was not reliably successful in meeting its objective. In our experience, basal joint hemiarthroplasty with this prosthesis resulted in pain relief, restoration of function, and preservation of appearance in 135, 138, and 142 thumbs, respectively, at a mean followup of 72.1 months, and complications were few. Our results are superior to those achieved with previous implant types and designs. In our experience, compared with tendon interposition, the implant operative procedure is easier to perform (mean implant operative time, 44 minutes versus 63 minutes) and the recovery time is shorter (mean, 12 weeks versus 22 weeks). Because the prosthesis used in our study is more anatomically shaped than other prostheses, our patients had increased stability, rare subluxation, and rare intrusion of the implant into the remodeled trapezium. In addition, the modularity of the implant simplifies the surgical procedure and permits more options for achieving optimal fit at the time of implantation. Failed cases can be treated, if necessary, by implant removal, trapeziectomy, and tendon interposition. Our results support continued use of this procedure and implant in addition to the need for additional studies with longer followups.
